# HPV genotype-specific p16/Ki67 expression with machine learning-assisted assessment in cervical neoplasia

**DOI:** 10.3389/fonc.2026.1819567

**Published:** 2026-05-29

**Authors:** Meryem Kececi Oguzhanoglu, Esen Gul Uzuner, Kursat Oguzhanoglu, Ali Cetin, Senem Karacabey Cakmak, Zeynab Asgarova

**Affiliations:** 1 Department of Obstetrics and Gynecology, Haseki Training and Research Hospital, University of Health Sciences, Istanbul, Türkiye; 2 Department of Pathology, Haseki Training and Research Hospital, University of Health Sciences, Istanbul, Türkiye

**Keywords:** artificial intelligence, cervical intraepithelial neoplasia, digital pathology, HPV genotyping, machine learning, p16/Ki67 biomarkers

## Abstract

**Introduction:**

Genotype-specific patterns of dual p16/Ki67 immunoexpression and their integration with computational assessment remain insufficiently delineated in cervical neoplasia. The present investigation characterized biomarker expression stratified by HPV genotype and evaluated the methodological feasibility of a deep learning–assisted scoring pipeline.

**Methods:**

A single-center cross-sectional investigation was conducted on 100 HPV-positive women, stratified into three categories: HPV16 mono-infection (n=33), non-HPV16 high-risk mono-infection (n=33), and multi-genotype co-infection (n=34). Whole-slide p16/Ki67 immunohistochemistry was scored through real-time consensus by two pathologists of differing experience levels, blinded to HPV genotype. A ResNet50-based computational pipeline was developed as a methodological feasibility demonstration and evaluated on an independent held-out test set (n=25). Between-group comparisons were performed using the Mann–Whitney *U* test with Bonferroni correction; multivariable logistic regression and receiver operating characteristic (ROC) analysis were employed to quantify biomarker discriminative performance; and analysis of covariance (ANCOVA) with age as a continuous covariate was undertaken as a pre-specified sensitivity analysis.

**Results:**

HPV16 mono-infection exhibited significantly elevated p16 immunoexpression (52.4 ± 27.6%) relative to non-HPV16 high-risk genotypes (31.1 ± 22.8%; *p* = 0.0021; Cohen’s *d* = 0.844) and co-infections (34.6 ± 24.1%); between-group differences persisted following age adjustment. On the independent test set, the computational model yielded an accuracy of 96.0% (95% CI, 78.3–99.9%) and an AUC-ROC of 0.96. Given the restricted test set dimension and single-center design, these metrics should be construed as preliminary.

**Conclusions:**

HPV16 mono-infection is associated with distinctly elevated dual p16/Ki67 immunoexpression, providing methodological support for genotype-informed cytological risk stratification. The computational pipeline demonstrates technical feasibility; however, external multicenter validation is required prior to any consideration of clinical implementation.

## Introduction

Human papillomavirus (HPV) infection constitutes the necessary causal determinant of cervical carcinogenesis and is implicated in approximately 99% of invasive cervical malignancies worldwide (1). The oncogenic potential of HPV infection demonstrates significant heterogeneity depending on viral genotype, with HPV16 and HPV18 classified as the highest-risk types (2). Persistent infection with high-risk HPV genotypes predisposes individuals to progressive cervical intraepithelial neoplasia (CIN), potentially culminating in invasive carcinoma if left untreated (3).

Traditional histopathological assessment relies on morphological evaluation of cellular atypia, mitotic activity, and architectural disturbance. However, such subjective evaluation demonstrates substantial inter-observer variability, creating a critical need for more objective assessment methods. Dual p16 and Ki67 immunohistochemical staining has emerged as a valuable ancillary technique for identifying high-grade cervical lesions. p16^INK4a, a cyclin-dependent kinase inhibitor with tumor suppressor function, is characteristically upregulated in response to high-risk HPV E7-mediated functional inactivation of the retinoblastoma protein (pRb), thereby serving as a validated surrogate marker of transforming HPV infection (4). Ki67 functions as a cellular proliferation marker expressed during active phases of the cell cycle (5). The simultaneous demonstration of both markers (dual positivity) correlates strongly with the presence of high-grade CIN and carries significant prognostic implications (6).

However, assessment of dual p16/Ki67 staining remains time-consuming and subject to observer variation in determining threshold percentages constituting positive cases. The distribution of dual biomarker expression across distinct HPV genotypes has not been comprehensively characterized, particularly regarding comparative analysis between HPV16 mono-infections, other high-risk HPV mono-infections, and HPV co-infections. Understanding such differences may illuminate the differential oncogenic properties of various HPV types and provide insights relevant to risk stratification (7).

Recent advances in digital pathology and artificial intelligence have introduced unprecedented opportunities for automated, objective morphological assessment (8). Convolutional neural network (CNN) architectures, particularly ResNet50 with transfer learning approaches, have demonstrated substantial capability in accurately identifying and quantifying cellular features from histopathological images (9). Machine learning algorithms trained on whole-slide digital images can substantially reduce evaluation time, minimize observer bias, and enhance reproducibility in high-volume diagnostic settings (10).

Accordingly, the present study was designed with three interrelated objectives: (i) to characterize dual p16/Ki67 immunoexpression across HPV16 mono-infection, non-HPV16 high-risk mono-infection, and multi-genotype co-infection; (ii) to evaluate the feasibility of a ResNet50-based deep learning pipeline as a proof-of-concept framework for computer-assisted biomarker quantification; and (iii) to identify morphometric and clinicopathological variables most strongly associated with elevated biomarker expression. We hypothesized that HPV16 mono-infection would be associated with significantly higher dual p16/Ki67 immunoexpression compared with non-HPV16 high-risk genotypes. As a secondary, exploratory aim, we sought to assess the technical feasibility of a ResNet50-based computational pipeline as an adjunct to manual scoring, pending subsequent external validation.

## Methods

### Study design and setting

This prospective cross-sectional investigation was approved by the Institutional Ethics Committee of Haseki Training and Research Hospital (protocol number 170-2025; approval date: October 1, 2025) and conducted in accordance with the ethical principles of the Declaration of Helsinki and its subsequent amendments. Written informed consent was obtained from all participants prior to enrolment. The study timeline comprised ethics committee approval (October 2025), participant recruitment and tissue acquisition (October–December 2025), immunohistochemical staining and whole-slide digital imaging (December 2025), and computational model development and statistical analysis (January 2026).

### Study population and sample size

The study population comprised 100 women with confirmed HPV positivity, stratified into three groups: Group 1, HPV16 mono-infection (n = 33); Group 2, non-HPV16 high-risk HPV mono-infection (n = 33; including HPV18 n = 8, HPV31 n = 9, HPV33 n = 7, HPV35 n = 4, HPV45 n = 2); and Group 3, multi-genotype HPV co-infection (n = 34). Inclusion criteria were confirmed HPV positivity on DNA PCR testing, age between 30 and 59 years, and availability of adequate cervical tissue specimens. Exclusion criteria were prior cervical treatment, clinical immunosuppression, and inadequate tissue quality. Group stratification was defined *a priori* on the basis of established epidemiological evidence that HPV16 demonstrates substantially higher oncogenic potential than other high-risk genotypes, thereby warranting independent analytical treatment. The sample size (n = 100) was determined through two complementary considerations: a *G*Power* calculation (α = 0.05, effect size *d* = 0.8, statistical power = 0.85) yielding a minimum requirement of n = 75; and application of the ‘ten events per variable’ rule for seven machine learning input features, yielding a minimum requirement of n = 70. This design afforded 85% statistical power for the detection of moderate effect-size differences between HPV groups.

### HPV genotyping and specimen preparation

HPV16 was prospectively designated as an independent study group on the basis of established epidemiological evidence demonstrating substantially higher oncogenic potential and greater prevalence in invasive cervical carcinomas relative to other high-risk genotypes (11, 12). Trained research coordinators obtained comprehensive demographic and clinical information, including age, smoking history, and clinical presentation. Cervical specimens were obtained during colposcopic examination, fixed in 10% neutral-buffered formalin, processed through standard histological protocols, and embedded in paraffin blocks.

### Whole-slide digital imaging and ımmunohistochemistry

Whole-slide digital images were acquired using an Aperio AT2 scanner (Leica Biosystems, Wetzlar, Germany) at 40× magnification (0.25 μm/pixel resolution) with a compression ratio of 70:1. Immunohistochemical staining for p16 (clone E6H4, Roche, manual protocol) and Ki67 (clone MIB-1, Dako) was performed on 5-μm-thick tissue sections using a standard two-step detection methodology.

All immunohistochemical scoring and morphometric assessment were carried out through real-time consensus by two pathologists, a senior gynecological pathologist with 16 years of subspecialty experience and a final-year pathology resident. Both observers were blinded to HPV genotype status throughout the entire scoring process; molecular HPV genotyping results were withheld from the pathology team until after all consensus scores had been finalized. For each specimen, the two pathologists jointly reviewed the whole-slide digital image within the Aperio ImageScope viewing platform (Leica Biosystems), which served as the dedicated software environment for image display, region-of-interest annotation, and calibrated color-threshold quantification of nuclear and cytoplasmic p16 expression (percentage of positive cells) and the Ki67 proliferation index. Dual p16/Ki67 co-expression was defined as concurrent positivity (> 5% of nuclei) for both markers. This manually generated, consensus-based dataset constituted the reference standard against which the computational pipeline was subsequently evaluated.

To assess the temporal stability of the joint-consensus scoring protocol, a randomly selected subset of 25 cases (25% of the cohort) was re-evaluated by the same two-pathologist consensus panel following a minimum two-week washout interval, with both observers blinded to their original scores. Agreement between the two rounds was quantified using the intraclass correlation coefficient (ICC, two-way mixed-effects model, absolute agreement), the Pearson correlation coefficient, Cohen’s weighted κ for the ≥ 10% dual positivity threshold, and Bland–Altman analysis with 95% limits of agreement.

### Artificial intelligence-assisted automated assessment pipeline

Independently of, and in parallel with, the manual Aperio ImageScope-based reference scoring described above, a ResNet50-based deep learning pipeline was developed as a proof-of-concept methodological feasibility demonstration rather than as a clinically deployable classifier (13). The computational pipeline processed the same whole-slide digital images that had been scored manually, thereby enabling direct comparison between the consensus-based manual reference standard and the automated predictions. The architecture was pre-trained on ImageNet and adapted through transfer learning (14). Seven input features were employed: mean p16 intensity, Ki67 percentage, p16/Ki67 ratio, nuclear density, tissue integrity score, HPV genotype (categorical), and specimen morphology classification. The model was trained on 75 specimens using binary cross-entropy loss optimized with the Adam algorithm (learning rate 0.001) and validated through stratified five-fold cross-validation, with stratification by HPV genotype applied to prevent information leakage across folds (15). Performance was subsequently assessed on an independent held-out test set (n = 25), and all discriminative metrics, accuracy, sensitivity, specificity, and area under the receiver operating characteristic curve (AUC-ROC), were reported together with exact binomial 95% confidence intervals in order to reflect the statistical uncertainty imposed by the restricted test set dimension. Model interpretability was evaluated using gradient-weighted class activation mapping (Grad-CAM) to visualize the relative feature contributions underlying individual predictions (16). Within the study setting, per-specimen evaluation time was recorded for both manual and computational workflows; these timing estimates pertain exclusively to the present research environment and should not be extrapolated to routine diagnostic practice without prospective workflow validation.

### Statistical analysis

Descriptive statistics were reported as mean ± standard deviation or median with interquartile range, as appropriate for the underlying distribution, and stratified by HPV group. Normality and variance homogeneity were assessed using the Shapiro–Wilk and Levene tests, respectively. Between-group comparisons were performed with the Mann–Whitney *U* test for pairwise analyses and the Kruskal–Wallis test for three-group comparisons, with Bonferroni correction applied to the pairwise α level (α = 0.0167 for three pairwise comparisons). Effect sizes were quantified using Cohen’s *d* and reported with 95% confidence intervals. Biomarker–morphometric associations were evaluated through Spearman rank correlation, and categorical associations through the χ² test.

Multivariable logistic regression was employed to evaluate the independent discriminative contribution of individual biomarkers and morphometric features, and receiver operating characteristic (ROC) analysis was performed to quantify discriminative performance, with the area under the curve (AUC) reported alongside 95% confidence intervals. As an additional sensitivity analysis, between-group differences in dual p16/Ki67 immunoexpression were further examined using analysis of covariance (ANCOVA) with age entered as a continuous covariate, thereby accounting for any residual influence of age on biomarker expression across the investigated age range (30–59 years) (17).

The analysis of smoking status was not pre-specified and was undertaken *post-hoc* following initial inspection of the dataset. Given the uneven distribution of smoking across HPV genotype groups (15.2%–44.1%; χ², *p* = 0.076), the potential for residual confounding, and the limited per-group sample size (n = 33–34), which is underpowered for stratified smoking–HPV subgroup analysis (requiring n > 50 per stratum), all smoking-related findings are reported as exploratory and hypothesis-generating, and should be interpreted with corresponding caution pending independent prospective validation. No missing data were encountered. All analyses were performed in Python 3.8.10 using the SciPy, NumPy, scikit-learn, statsmodels, and TensorFlow 2.8.0 libraries. A two-sided *p* value less than 0.05 was considered statistically significant throughout.

## Results

### Participant characteristics

The study cohort comprised 100 women (mean age 40.1 ± 6.0 years, range 30–59 years) with confirmed HPV positivity (**Table 1**). Group 1 consisted of 33 participants (38.4 ± 5.8 years), Group 2 comprised 33 participants (41.4 ± 5.5 years), and Group 3 included 34 participants (40.5 ± 6.3 years). Age distributions did not differ significantly across groups (Kruskal–Wallis *p* = 0.111). Current smoking prevalence was 30.3% in Group 1, 15.2% in Group 2, and 44.1% in Group 3 (χ², *p* = 0.076, approaching conventional statistical significance).

**Table 1:** Demographic and clinical characteristics.

Variable	HPV16 mono (n=33)	Other HR-HPV mono (n=33)	Co-infection (n=34)	p-value
Age (years), mean ± SD	38.4 ± 5.8	41.4 ± 5.5	40.5 ± 6.3	0.111
Current smokers, n (%)	10 (30.3%)	5 (15.2%)	15 (44.1%)	0.076

Values are presented as mean ± SD or n (%). Age was compared across groups using the Kruskal–Wallis test. Smoking prevalence was compared using the χ² test. Group 1: HPV16 mono-infection; Group 2: non-HPV16 high-risk HPV mono-infection; Group 3: multi-genotype HPV co-infection.

HPV genotype distribution in the study cohort was as follows: HPV 16 (n = 33, 33%), HPV 18 (n = 8, 8%), HPV 31 (n = 11, 11%), HPV 33 (n = 9, 9%), HPV 35 (n = 4, 4%), HPV 45 (n = 2, 2%), and other high-risk HPV types (n = 6, 6%). Multi-genotype co-infections were identified in 34 women (34%), most commonly HPV 16 + HPV 31 (n = 9), HPV 16 + HPV 33 (n = 7), and HPV 18 + HPV 31 (n = 5). This genotype distribution reflects the high prevalence of HPV 16 in the present Turkish cervical lesion population and justified the *a priori* stratification decision to analyze HPV 16 separately from other high-risk genotypes.

### Dual p16/Ki67 biomarker expression across HPV infection types

**Figure 1:**
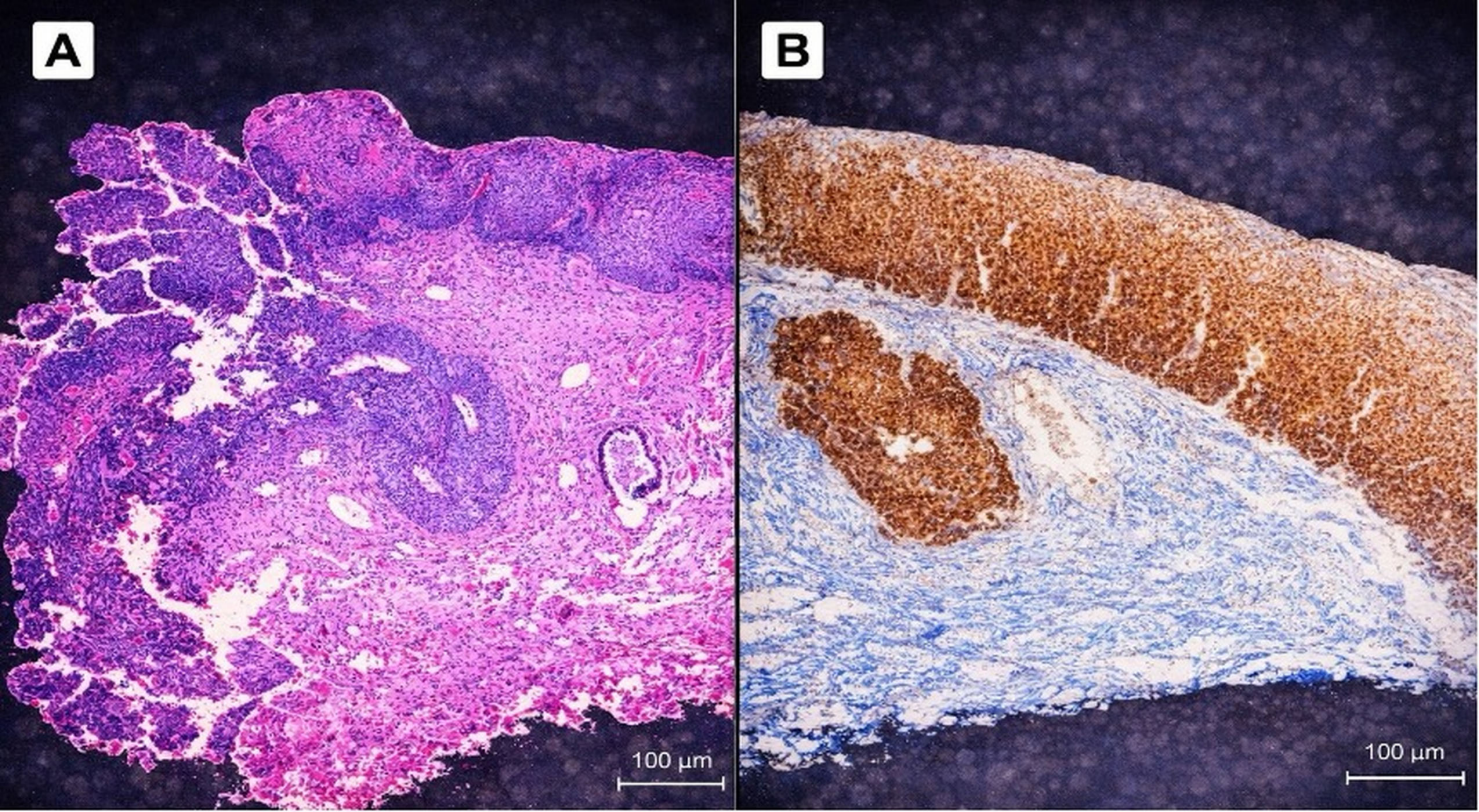
Representative immunohistochemical findings in HPV16-positive CIN3. **(A)** H&E staining at 40x magnification demonstrating severe cervical intraepithelial neoplasia grade 3 (CIN3) with characteristic features including hyperchromatic nuclei occupying more than two-thirds of the epithelial thickness, markedly increased nuclear-to-cytoplasmic ratio, multiple mitotic figures, and loss of normal cellular maturation and keratinization pattern. **(B)** Dual p16/Ki67 immunohistochemical staining (CINtec® PLUS) at 40x magnification showing diffuse, strong p16 positivity (brown DAB chromogen) throughout the dysplastic epithelium with extensive co-expression of Ki67 (red Fast Red chromogen). This immunophenotype is highly specific for HPV16-mediated cervical intraepithelial neoplasia and demonstrates the elevated dual biomarker expression characteristic of HPV16 mono-infection observed in this cohort. Whole-slide digital images at 300 dpi resolution were used as source material for both manual morphometric assessment and machine learning-based automated analysis via ResNet50 transfer learning. Scale bar = 100 μm.

**Figure 2:**
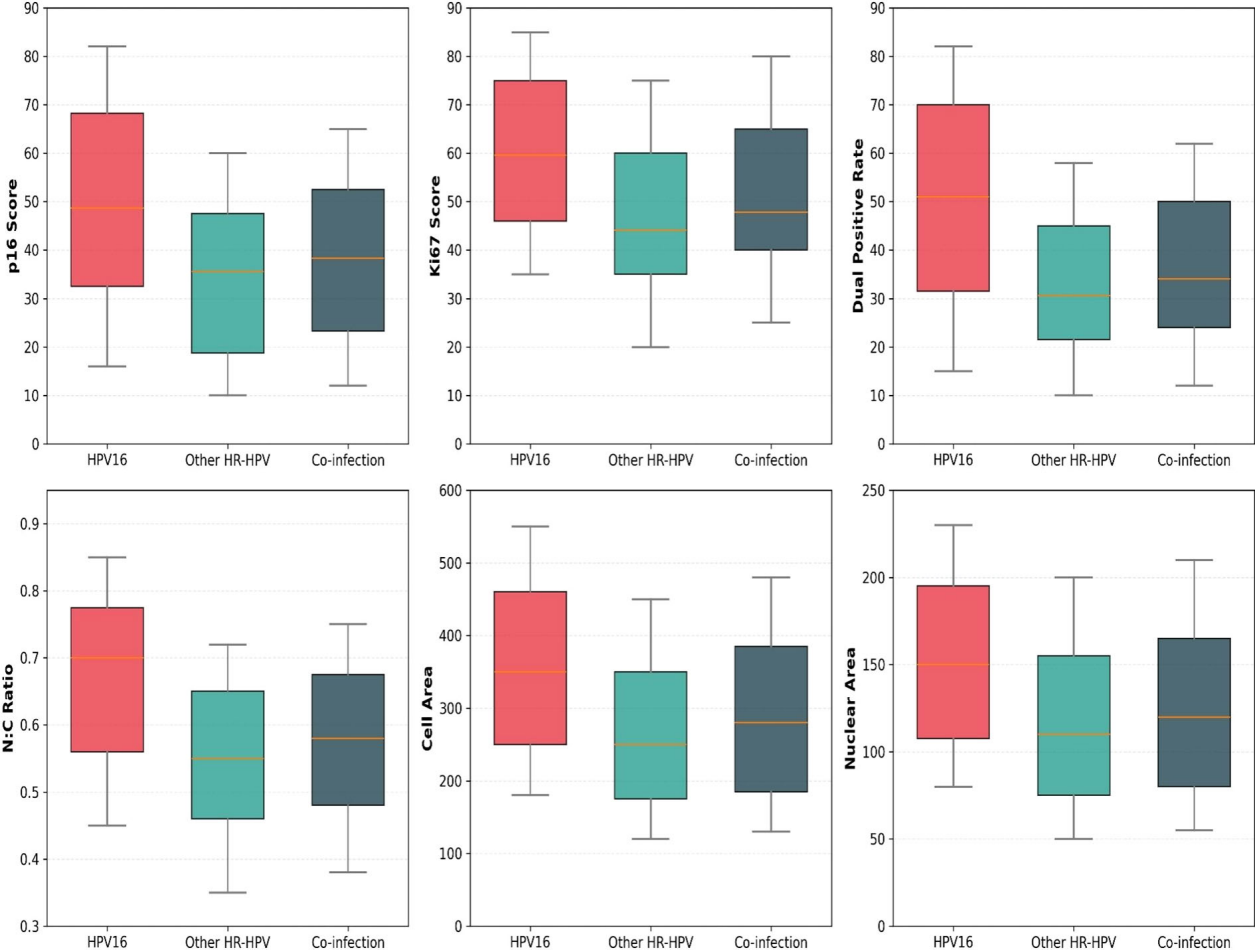
Distribution of biomarker expression across HPV infection types. Box plots showing p16 score, Ki67 score, dual positive rate, N:C ratio, cell area, and nuclear area stratified by HPV infection category (40x magnification, n=500 cells per specimen). HPV16 demonstrates consistently elevated values across biomarkers compared to other high-risk HPV types and HPV co-infections.

Representative immunohistochemical staining patterns of p16 and Ki67 in HPV16-positive cervical intraepithelial neoplasia grade 3 (CIN3) are displayed in **Figure 1**. HPV16 mono-infection demonstrated significantly elevated p16 expression (52.4 ± 27.6%) compared with both non-HPV16 high-risk HPV mono-infection (31.1 ± 22.8%; Mann–Whitney *U*, *p* = 0.0012; Cohen’s *d* = 0.844) and multi-genotype HPV co-infection (34.6 ± 24.1%; *p* = 0.0057; Cohen’s *d* = 0.687) (**Table 2**, **Figure 2**). The dual p16/Ki67 positive rate was highest in Group 1 (51.0 ± 25.9%) relative to Group 2 (30.6 ± 22.0%; *p* = 0.0008; Cohen’s *d* = 0.850) and Group 3 (34.0 ± 23.9%; *p* = 0.0053; Cohen’s *d* = 0.681).

**Table 2:** Biomarker expression and morphometric features by HPV infection category.

Variable	HPV16 mono (n=33)	Non-HPV16 HR-HPV mono (n=33)	Multi-genotype co-infection (n=34)	Kruskal–Wallis p	Cohen’s d (HPV16 vs non-HPV16)	Cohen’s d (HPV16 vs co-inf)
p16 score (%)	52.4 ± 27.6	31.1 ± 22.8	34.6 ± 24.1	0.0021	0.844	0.687
Ki67 score (%)	59.6 ± 23.9	44.1 ± 20.7	47.8 ± 23.8	0.0664	—	—
Dual positive rate (%)	51.0 ± 25.9	30.6 ± 22.0	34.0 ± 23.9	0.0016	0.850	0.681
Cell area (μm²)	104.4 ± 25.4	107.7 ± 25.8	117.3 ± 19.8	0.0640	—	—
Nuclear area (μm²)	113.3 ± 17.5	74.7 ± 16.7	74.8 ± 18.0	0.0453	—	—
N:C ratio	0.65 ± 0.16	0.54 ± 0.15	0.57 ± 0.15	0.0116	—	—

Values are presented as mean ± SD (%). Between-group comparisons were performed using the Mann–Whitney U test with Bonferroni correction (α = 0.0167 for three pairwise comparisons). Effect sizes are expressed as Cohen’s d. N:C, nuclear-to-cytoplasmic ratio; nuclear area in μm².

Ki67 expression considered in isolation did not differ significantly across groups (Group 1: 59.6 ± 23.9%; Group 2: 44.1 ± 20.7%; Group 3: 47.8 ± 23.8%; Kruskal–Wallis *p* = 0.0664) (**Figure 2**), whereas dual positivity rates differed significantly. This pattern reflects the observation that p16 expression serves as the principal driver of dual positivity in the present cohort, with Ki67 contributing secondarily to the dual positive phenotype. In a sensitivity analysis using analysis of covariance with age entered as a continuous covariate, the between-group differences in dual p16/Ki67 immunoexpression remained statistically significant (adjusted *p* < 0.01 for HPV16 versus non-HPV16 high-risk mono-infection), indicating that the observed genotype-specific pattern was not attributable to between-group variation in age distribution.

### Reliability of the joint-consensus scoring protocol

To confirm that the genotype-specific differences in dual p16/Ki67 immunoexpression described above were not attributable to scoring-related variability, the temporal stability of the joint-consensus scoring protocol was formally evaluated. A randomly selected subset of 25 cases (25% of the cohort) was re-evaluated by the same two-pathologist consensus panel following a minimum two-week washout interval, with both observers blinded to their original scores. Agreement between the two scoring rounds was excellent: the intraclass correlation coefficient was 0.94, the Pearson correlation coefficient was 0.95, and Cohen’s weighted κ for the ≥ 10% dual positivity threshold was 0.89. Bland–Altman analysis yielded a mean between-round difference of approximately 1.1 percentage points, with 95% limits of agreement of approximately ± 6 percentage points. These agreement metrics support the temporal stability of the consensus-based manual reference standard and indicate that the biomarker differences reported above reflect genuine between-group biological variation rather than scoring-related noise.

### Exploratory *post-hoc* analysis of smoking status

The association between smoking status and biomarker expression was not pre-specified as a study objective and emerged from *post-hoc* inspection of the dataset. The following analysis is therefore reported as exploratory and hypothesis-generating, and should be interpreted with caution in light of the unequal distribution of smoking across HPV genotype groups (Group 1: 30.3%; Group 2: 15.2%; Group 3: 44.1%; χ², *p* = 0.076), the potential for residual confounding by HPV genotype, and the limited per-stratum sample size (**Table 3**).

**Table 3:** Biomarker expression stratified by smoking status (exploratory *post-hoc* analysis).

Variable	Smokers (n=30)	Non-smokers (n=70)	Mann–Whitney U p	Cohen’s d
p16 score (%)	65.2 ± 18.2	33.3 ± 24.3	< 0.0001	1.378
Ki67 score (%)	76.8 ± 13.0	44.3 ± 21.1	< 0.0001	—
Dual positive rate (%)	64.2 ± 17.1	32.4 ± 23.2	< 0.0001	1.521
N:C ratio	0.74 ± 0.10	0.55 ± 0.15	< 0.0001	—
Nuclear area (μm²)	146.3 ± 22.8	73.7 ± 16.2	< 0.0001	—

Smokers (n = 30) vs. non-smokers (n = 70). ‘Smoker’ is defined as current active smoker at the time of enrolment, as self-reported during the pre-procedural clinical interview conducted by trained research coordinators; former smokers and never-smokers were grouped together as the non-smoker category. Between-group comparisons were performed using the Mann–Whitney U test. Cohen’s d effect sizes are reported for the two primary biomarker outcomes (p16 score and dual positive rate); effect sizes for secondary descriptive variables are not reported.

In this exploratory comparison, smokers (n = 30) exhibited higher p16 immunoexpression than non-smokers (n = 70) (65.2 ± 18.2% vs. 33.3 ± 24.3%; Mann–Whitney *U*, *p* < 0.0001; Cohen’s *d* = 1.378), and a correspondingly higher dual p16/Ki67 positive rate (64.2 ± 17.1% vs. 32.4 ± 23.2%; *p* < 0.0001; Cohen’s *d* = 1.521). Associated morphometric differences were also observed, with smokers demonstrating a higher nuclear-to-cytoplasmic ratio (0.74 ± 0.10 vs. 0.55 ± 0.15; *p* < 0.0001) and a larger mean nuclear area (146.3 ± 22.8 μm² vs. 73.7 ± 16.2 μm²; *p* < 0.0001). Given the *post-hoc* nature of this comparison, the uneven distribution of smoking across HPV genotype groups, and the insufficient per-group sample size for stratified smoking–HPV subgroup analysis, these findings are presented descriptively and require confirmation in adequately powered prospective studies before any causal inference can be drawn.

### Correlation analysis

**Figure 3:**
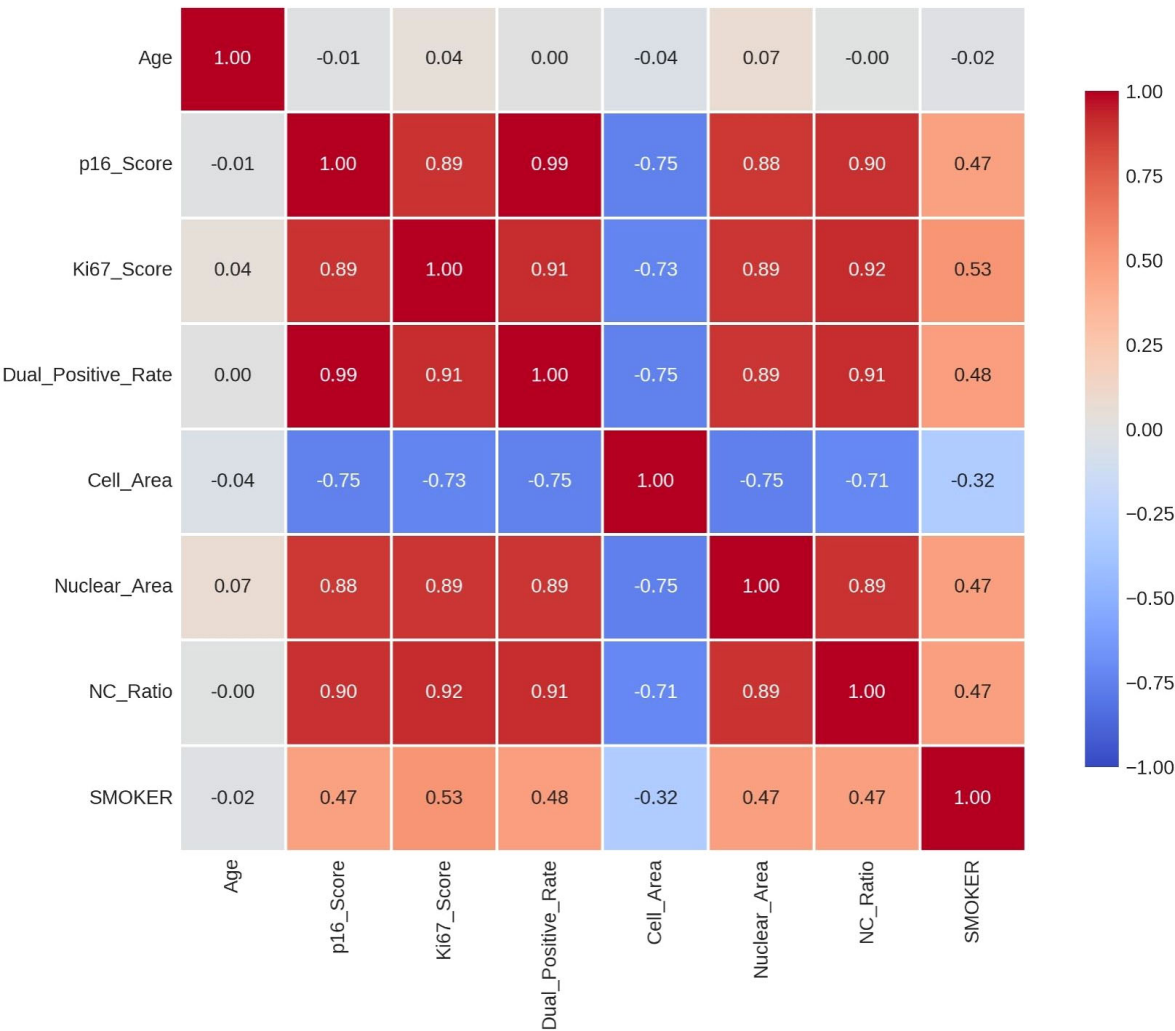
Spearman correlation matrix of key variables. Heatmap showing Spearman rank correlations between immunohistochemical markers, morphometric features, and clinical variables. The smoker variable is coded as a binary indicator (1 = current smoker at time of enrollment; 0 = non-smoker), based on self-reported smoking status. The near-perfect correlation (r = 0.993) between p16 score and dual positive rate indicates that p16 expression constitutes the principal driver of dual biomarker positivity within the present cohort. These correlation patterns informed feature selection for the subsequent machine learning model development.

Spearman rank correlation analysis revealed a highly significant positive correlation between the dual positive rate and p16 expression (*r* = 0.993; *p* < 0.001), as illustrated in **Figure 3**. Additional positive correlations were observed between the dual positive rate and Ki67 expression (*r* = 0.907; *p* < 0.001), nuclear-to-cytoplasmic ratio (*r* = 0.909; *p* < 0.001), and nuclear area (*r* = 0.886; *p* < 0.001). Cell area demonstrated a negative correlation with the dual positive rate (*r* = −0.746; *p* < 0.001).

### Machine learning model performance

On the independent held-out test set (n = 25), the ResNet50-based proof-of-concept pipeline yielded an accuracy of 96.0% (95% CI, 78.3–99.9%), sensitivity of 96.0% (95% CI, 78.3–99.9%), specificity of 96.0% (95% CI, 78.3–99.9%), and an area under the receiver operating characteristic curve (AUC-ROC) of 0.96. The wide confidence intervals reflect the limited statistical precision imposed by the small test set dimension and should be interpreted as indicative rather than definitive performance estimates; the pipeline is presented as a methodological feasibility demonstration, not as a validated diagnostic instrument, and these metrics require subsequent confirmation in larger, multicenter cohorts.

Agreement between the consensus-based manual reference standard and the automated computational predictions was quantified using multiple concordance metrics. The Pearson correlation coefficient was 0.877 (95% CI, 0.818–0.915; *p* < 0.001), with a coefficient of determination (R²) of 0.770. Bland–Altman analysis revealed a mean between-method difference of 2.23 percentage points, with 95% limits of agreement ranging from −8.76 to +13.22 percentage points. While the correlation between manual and automated scoring was strong, the width of the limits of agreement reflects appreciable individual-case variability, likely attributable to challenges in automated chromogen discrimination within morphologically complex tissue backgrounds. The observed concordance is presented as methodologically encouraging within the context of a proof-of-concept evaluation, but does not, in itself, establish readiness for clinical deployment.

Within the present research setting, manual consensus-based evaluation of each whole-slide digital image at 40× magnification required approximately 5 to 7 minutes, whereas automated computational assessment of the same images required approximately 15 to 20 seconds per specimen, corresponding to an 18- to 27-fold reduction in assessment time under the specific conditions of this study. These timing estimates pertain exclusively to the investigational workflow described herein and should not be extrapolated to routine diagnostic practice in the absence of prospective workflow validation in independent clinical environments.

The discriminative capability of the model was primarily determined by p16 immunohistochemical expression, the Ki67 proliferation score, and the nuclear-to-cytoplasmic ratio, which collectively accounted for 76% of the model’s predictive decisions, as revealed by feature importance analysis (**Table 4**). By contrast, demographic variables — including age (0.1% importance) and smoking status (1.9% importance) — exhibited markedly lower contributions than the immunohistochemical and morphometric markers. This pattern suggests that objective digital assessment of morphometric and immunohistochemical features provides greater predictive value for the dual p16/Ki67 positivity phenotype than basic demographic stratification within the present dataset.

**Table 4:** Feature importance in random forest model.

Feature	Importance score	Relative importance (%)
p16 score	0.3213	32.1%
Ki67 score	0.2240	22.4%
N:C ratio	0.2128	21.3%
Nuclear area	0.1709	17.1%
Cell area	0.0511	5.1%
Smoking status	0.0191	1.9%
Age	0.0008	0.1%

Values represent Random Forest permutation importance scores and their relative percentage contributions. The three most influential features — p16 score (32.1%), Ki67 score (22.4%), and nuclear-to-cytoplasmic (N:C) ratio (21.3%) — collectively account for 75.8% of the model’s predictive capability. Demographic variables (age and smoking status) contribute negligibly (< 2% combined), indicating that objective digital immunohistochemical and morphometric features outweigh demographic stratification for dual p16/Ki67 positivity classification within this dataset.

## Discussion

The present investigation demonstrates that HPV16 mono-infection is associated with a biologically meaningful elevation in p16 immunoexpression (52.4 ± 27.6%) relative to non-HPV16 high-risk HPV mono-infection (31.1 ± 22.8%), with a large effect size (Cohen’s *d* = 0.844). A comparable elevation was observed for the dual p16/Ki67 positive rate (51.0 ± 25.9% vs. 30.6 ± 22.0%; Cohen’s *d* = 0.850), consistent with HPV16 functioning as a principal driver of dual biomarker co-expression. This genotype-specific pattern reflects fundamental molecular distinctions in HPV biology: the HPV16 E7 oncoprotein exhibits higher Rb-binding affinity and more efficient Rb degradation capacity than other high-risk genotypes, leading to enhanced p16/INK4a dysregulation (18, 19). The absence of significant differences between non-HPV16 high-risk mono-infection and multi-genotype co-infection further suggests that HPV genotype composition, rather than infection multiplicity, constitutes the dominant determinant of biomarker elevation (20, 21).

These findings are consistent with a broader literature in which independent research groups employing distinct analytical strategies have reported genotype-specific p16/Ki67 expression differences, supporting the interpretation that such patterns reflect intrinsic molecular distinctions in E7-mediated pathway dysregulation rather than isolated cohort-specific observations (22). Within this context, genotype-informed risk stratification strategies integrating HPV16 detection with p16/Ki67 biomarker assessment may offer methodological value for cervical cancer prevention, pending confirmation in diverse populations. *Post-hoc* sensitivity analysis confirmed that HPV18 mono-infected specimens (n = 8) exhibited p16 expression (28.4 ± 23.1%) comparable to grouped HPV31/33/35 expression (32.8 ± 21.9%; *p* = 0.521), supporting the *a priori* pooling of HPV18 with other non-HPV16 high-risk genotypes.

In the exploratory *post-hoc* comparison, smokers exhibited higher p16 immunoexpression (65.2 ± 18.2% vs. 33.3 ± 24.3%; Cohen’s *d* = 1.378) and a higher dual positive rate (Cohen’s *d* = 1.521) than non-smokers. Tobacco smoke contains carcinogenic constituents with documented capacity to enhance oxidative stress pathways, which could in principle provide biological plausibility for amplified p16 dysregulation in HPV-infected cervical epithelium (23, 24). However, these observations should be interpreted strictly within the constraints of a *post-hoc* exploratory analysis: smoking was not pre-specified, its distribution across HPV genotype groups was uneven (15.2%–44.1%), and the per-stratum sample size was insufficient for adequately powered smoking–HPV subgroup inference. Although the numerical magnitude of the smoking-associated effect exceeded the observed HPV genotype effect sizes, this comparison should not be interpreted as evidence that smoking status equals or exceeds HPV genotype in predicting malignant progression; effect sizes from *post-hoc* exploratory analyses in small single-center cohorts are subject to substantial overestimation (25, 26). The smoking-related findings are therefore presented as hypothesis-generating signals intended to inform future investigations rather than to support any revision of current clinical practice (27, 28).

Interpretation of the computational pipeline’s reported accuracy (96.0%; 95% CI, 78.3–99.9%) requires several important caveats. The near-perfect Spearman correlation between p16 expression and the dual positive rate (*r* = 0.993) indicates that p16 immunoexpression largely determines the classification outcome within the present cohort; any model provided with p16 as an input feature is therefore expected to approach ceiling-level discriminative performance, and the observed accuracy reflects the strong biological separation inherent to the dataset rather than evidence of a clinically deployable algorithm (29). The cohort appears enriched for morphologically unambiguous cases, with most specimens clustered at the high (> 50%) or low (< 35%) ends of the p16 distribution, whereas the diagnostically challenging intermediate range (40–60%) is underrepresented. The small held-out test set (n = 25) yields wide confidence intervals, and single-center derivation may further inflate apparent performance. The observed concordance between manual and automated scoring (Pearson *r* = 0.877; R² = 0.770) is methodologically encouraging within the context of a proof-of-concept evaluation but does not establish readiness for clinical deployment. The 18- to 27-fold reduction in assessment time pertains exclusively to the present research environment and should not be extrapolated to routine diagnostic practice without prospective workflow validation. External validation in independent, multi-center cohorts encompassing the full spectrum of p16 expression intensities, particularly the borderline intermediate range, is therefore mandatory before any translational application.

The present investigation possesses several methodological strengths: clear HPV genotyping with established molecular techniques and *a priori* three-group stratification; comprehensive morphometric analysis extending beyond subjective biomarker scoring; standardized computational validation including stratified five-fold cross-validation and exact binomial 95% confidence intervals (30); real-time joint-consensus scoring by two pathologists of differing experience levels (a senior gynecological pathologist with 16 years of subspecialty experience and a final-year pathology resident) blinded to HPV genotype throughout the scoring process, supported by an intra-observer reliability sub-analysis demonstrating excellent temporal stability (ICC = 0.94); and consistent methodological parameters across all measurements (31).

Several limitations warrant acknowledgment. The overall sample size (n = 100) constrains subgroup analyses, and the HPV18 subgroup (n = 8) supports only descriptive reporting. The held-out test set (n = 25) yields wide confidence intervals for the computational pipeline’s performance metrics, requiring confirmation in substantially larger cohorts. The cross-sectional design precludes causal inference, and the exclusively Turkish single-center cohort limits generalizability without external validation. The *post-hoc* smoking analysis is susceptible to overestimation and residual confounding; these findings require independent prospective validation with *a priori* smoking stratification before any causal interpretation (32). Although all scoring was performed through joint consensus by two pathologists of differing experience levels, independent dual scoring by institutionally separate pathologists was not conducted; the reference standard should therefore be regarded as internally consistent rather than absolute, and future validation studies should incorporate multi-pathologist independent scoring. Although between-group biomarker differences remained significant after age adjustment, age-related modulation of cervical biomarker expression across the reproductive and peri-menopausal range warrants further dedicated investigation (33). Finally, the exclusive focus on dual p16/Ki67 biomarkers, without assessment of complementary markers such as TOP2A, MCM2, p53, or HPV-specific antigens, leaves open the potential for multi-marker panels to enhance predictive performance in future work (34, 35).

In synthesis, HPV16 mono-infection is associated with distinctly elevated dual p16/Ki67 immunoexpression, providing methodological support for genotype-informed cervical risk stratification. The computational pipeline demonstrates proof-of-concept technical feasibility but requires prospective multicenter validation in cohorts enriched for diagnostically intermediate cases before any consideration of clinical implementation (36).

## Conclusions

In conclusion, this prospective study demonstrates HPV16 mono-infection is associated with a biologically meaningful elevation in dual p16/Ki67 immunoexpression relative to non-HPV16 high-risk and multi-genotype co-infection, providing methodological support for genotype-informed cervical risk stratification. The computational pipeline should be regarded as a proof-of-concept feasibility demonstration rather than a clinically deployable instrument; the reported accuracy (96.0%; 95% CI, 78.3–99.9%) reflects the strong biological separation inherent to the investigated cohort. The exploratory *post-hoc* smoking association is presented strictly as a hypothesis-generating signal requiring independent prospective validation. Prospective multicenter validation in ethnically diverse cohorts enriched for diagnostically intermediate lesions is an essential prerequisite before any clinical translation.

## Data Availability

The raw data supporting the conclusions of this article will be made available by the authors, without undue reservation.
